# T-cell acute lymphoblastic leukaemia: subtype prevalence, clinical outcome, and emerging targeted treatments

**DOI:** 10.1038/s41375-025-02599-2

**Published:** 2025-04-17

**Authors:** Maxim Buckley, David T. Yeung, Deborah L. White, Laura N. Eadie

**Affiliations:** 1 Blood Cancer, Precision Medicine Theme, South Australian Health and Medical Research Institute, Adelaide, SA, Australia; 2 Adelaide Medical School, Faculty of Health and Medical Sciences, University of Adelaide, Adelaide, SA, Australia; 3 Hematology Department, Central Adelaide Local Health Network, Adelaide, SA, Australia

**Keywords:** Cancer genomics, Cancer genomics

## Abstract

T-cell Acute Lymphoblastic Leukaemia (T-ALL) is a high-risk hematological disease constituting ~20% of acute leukemias. To date, the only subtype recognized by the World Health Organization’s International Consensus Classification is early T-cell precursor ALL. To improve clinical outcomes, several studies have investigated and defined T-ALL genomic subtypes within cohorts of varied ages and geographical locations. These studies have also utilized differing analysis methods including whole transcriptome, exome, or genome sequencing as well as immunophenotyping and cytogenetic testing. As a result, there are significant differences in reported subtypes as well as the frequency at which each occurs. The reported clinical outcomes for specific genomic alterations also depend on patient demographics and treatment protocols. This review synthesizes the data from four T-ALL genomic landscape studies establishing consensus and highlighting differences, details clinical outcomes for the most common genomic alterations observed in T-ALL patients, and proposes novel avenues for future investigation and treatment.

## Introduction

Acute Lymphoblastic Leukaemia (ALL) is a genomically complex disease that is aggressive and frequently fatal in some cohorts [1, 2]. ALL can affect people of any age, however, around 80% of cases occur in pediatric patients [2]. While ALL is considered to be a single disease it can be subclassified into T- and B-cell ALL [1]; T-ALL contributes to 15–20% of ALL cases depending on age group [2]. Genomic subclassification of B-ALL is more established as compared with T-ALL, with several recently defined subtypes now incorporated into World Health Organization (WHO) and International Consensus Classification (ICC) systems for acute leukemia [3]. The characterization and clinical significance of these entities have been reviewed elsewhere [4]. In contrast, the subclassification of T-ALL has not yet been widely adopted into the clinic. Reflecting this uncertainty, the WHO only recognizes Early T-cell Precursor-ALL (ETP-ALL) as a distinct subtype [3]. The ICC additionally recognizes *BCL11B*-activated ETP-ALL, and not otherwise specified (NOS) T-ALL along with 8 provisional entities, several of which are discussed in this review (*TAL1/2*-rearranged (*TAL1/2*r), *TLX1*r, *TLX3*r, *HOXA*, *LMO1/2*r, *NKX2*r, *SPI1*r, basic helix loop helix) [5]. Notwithstanding formal T-ALL subclassification systems, there is accumulating data to enhance a deeper understanding of the biology and genomics of T-ALL.

Patients with T-ALL usually have one or two driver genomic alterations capable of causing leukemic transformation, however, unique combinations of co-occurring mutations continue to be identified [6]. Despite the identification of recurrent potentially clinically targetable alterations including *NOTCH1* mutations [7]*, KMT2A* rearrangements (*KMT2A*r) [8], and the *NUP214::ABL1* gene fusion [9], T-ALL patients are routinely treated with intensive chemotherapy regardless of genotype [10]. Where possible, stem cell transplant is routinely utilized in adult patients [10] however, transplants are less frequent in pediatric patients [11]. Considerable research into alternative treatment options has occurred in the B-ALL sphere such as Bispecific T-cell engagers (BiTEs) [12] and chimeric antigen receptor (CAR-T) cells [13] however, innovative therapies for treatment resistant T-ALL patients are currently lacking and those who relapse or are treatment refractory will likely die of their disease.

Traditional, widely utilized methods of subclassification such as immunophenotyping and cytogenetics have yielded several clinically significant subtypes. However, through various next-generation genomic approaches, an increasing number of genomic subtypes of T-ALL have been identified that undoubtedly influence patient response to treatment [14–17]. These subtypes carry inherent risk and drug sensitivities, however, incorporating the knowledge gained through next-generation sequencing into clinical care presents a major challenge. Also, the systematic identification of druggable targets and effective rational therapies require robust pre-clinical evidence of underlying biology and function, drug efficacy and a comprehensive investigation of other factors that influence response and resistance, including the interplay of novel fusion genes with co-occurring mutations. For example, the efficacy of γ-secretase inhibitors and anti-Notch monoclonal antibodies for *NOTCH1* overexpressing cancers have been assessed in vitro and in vivo with some success [18] but severe gastrointestinal side effects and lack of efficacy in *NOTCH*-mutant breast cancers raised concerns about their clinical utility [19–21]. The chemotherapeutic nelarabine is approved for relapsed/refractory T-ALL patients in many countries, and additionally for newly diagnosed T-ALL patients in several others, but beyond this, limited treatment options exist [22]. Despite the variety of genomic drivers of disease offering potentially targetable therapeutic pathways, no such treatments are routinely used for T-ALL and represent a clear unmet need in the clinical setting.

This review covers the diagnostic journey of a T-ALL patient, discussing subtype identification, clinical outcomes and interrogating potential novel treatments. A patient who is feeling unwell may have a blood test ordered by their physician which, if leukaemia is the causative disease, will return an abnormal white blood cell count [23]. Following the initial test, a full blood count including leukocyte differential is ordered to determine blast cell percentage, this may also include morphological analysis of bone marrow [23].

ALL cannot be determined from morphology alone and further diagnostic testing such as flow cytometry is required to define B- or T-cell lineage ALL [23], and karyotyping and fluorescence in situ hybridization (FISH) to assess gross chromosomal abnormalities and specific chromosomal translocations, duplications, or deletions (e.g.: *ABL*-class, *KMT2A*-rearrangements). Specialized laboratories also conduct investigations not included in routine testing such as next generation sequencing analyses (transcriptomic, whole exome, and whole genome) and multiplex ligation-dependent probe amplification to detect cytogenetically cryptic genomic alterations and copy number variations in select genes relevant to the leukemic subtype [23]. Additional emerging techniques such as optical genome mapping, capable of detecting large scale structural variations, may prove useful as diagnostic tools in the future [24]. Furthermore, mRNA-sequencing provides insight on driving and co-occurring lesions (discussed later), specifically those amenable to targeted therapies with demonstrated efficacy in other diseases (e.g. FLT3 in Acute Myeloid Leukemia) [25].

We have identified four major T-ALL genomic subtype defining publications [14–17]. Though there is broad agreement on subtypes such as *MLLT10*r and *KMT2A*r, several genomic subtypes differ between these four papers due to variations in cohorts and methods of classification, summarized in Table 1. One of the first descriptions of the T-ALL genomic landscape came from the North American Children’s Oncology Group (264 children and young adults aged 1-29). Liu et al. utilized whole exome sequencing (WES) and transcriptomic sequencing to define 8 patient subtypes [15]. Two separate subsequent studies focussed on gene fusions and other structural alterations as the main subtype-defining lesions. The first, by Dai et al., was a large international cohort, (707 patients; 510 pediatric aged 1-17, 190 AYA/adults aged 18-75, 7 unknown) using mutational profiles and dysregulated gene expression signatures of leukemogenic factors to define 10 subtypes [14]. These findings were recapitulated by Müller et al. (131 patients; 6 pediatric, 62 adolescent and young adults (AYA), and 63 adults) using diagnostically available techniques including karyotyping and FISH [16]. The latest Children’s Oncology Group study by Pölönen et al. (1309 patients, including those reported in Liu et al. [15]; pediatric and AYA patients up to 30 years) grouped cases into 15 genomic subtypes based on whole genome (WGS), transcriptome (WTS), and WES analyses; the assay for transposase-accessible chromatin with sequencing (ATAC-seq) and sequencing chromosome conformation capture and interaction immunoprecipitation (HiChIP) were utilized for non-coding mutations and structural variations [17]. Details of the patient cohorts are described in Table 2.

**Table 1:** Genomic subtypes listed in T-ALL genomic landscape publications.

				Pölönen et al. 2024 [17]	Liu et al. 2017 [15]	*Dai et al. 2022 [14]	Muller et al. 2023 [16]	
			**Countries/regions cohort sourced from**	USA, Australia, Canada, New Zealand, Switzerland	USA, Australia, Canada, New Zealand, Switzerland	USA, Europe, Japan, Singapore, Malaysia, China	Germany	
			**Next generation sequencing techniques used**	WTS, WES, WGS	WTS, WES	WTS	Chromosome banding analysis, FISH, WGS, WTS	
			**Total patients**	1309	264	707	131	

Genomic subtype	Occurrence (rounded)	Genomic risk	Potential therapies	Subtype reported	Subtype reported	Subtype reported	Subtype reported	Comment
*LYL1/LMO2*	7% [15], 28% [14]	Low (pediatric) [15]High (AYA/adult) [116]	JAK inhibitors (ruxolitinib, tofacitinib, and upadacitinib) [123], JAK2 inhibitor (fedratinib) [125]	**Classifying driver alterations contributing to other subtypes:** 88/1309 *LMO2* TCR; 78/1309 *LMO2* enhancer SNV/Indel; 32/1309 *LMO2* enhancer gain; 21/1309 *LMO2* intergenic loss (*RAG2*); 15/1309 *LMO2* fusion; 14/1309 *LMO2* intergenic loss (*CAPRIN1*); 9/1309 *LMO2* other; 10/1309 *LYL1* fusion; 4/1309 *LYL1* other	*LYL1/LMO2* (n = 18) **Genetic driver alterations reported within subtype:** 4/18 *LMO2* fusion; 1/18 *LMO2* missense alteration; 3/18 *LMO2* copy number loss	G1		These cases were classified due to high expression of *LYL1* and/or *LMO2*. However, the drivers of this subtype likely include enhancer hijacking, enhancer mutations, mutations generating neoenhancers and intergenic inversions/deletions leading to deregulation of *LYL1/LMO2*. Alterations like these are only detectable by WGS.
*HOXA*	13% [15], 2% [14], 5% [16], 5% (3% *HOXA13*, 2%) [17]	Low (*HOXA9*::*TCR*) Intermediate (*HOXA13*) [17] High (*HOXA11*/*HOXA13*) [96]	-	*HOXA9* TCR (n = 21) **Genetic driver alterations reported within subtype:** 21/21 *HOXA9* TCR	*HOXA* (n = 33) **Genetic driver alterations reported within subtype:** 7/33 *HOXA10* fusion; 1/33 *HOXA10* copy number loss	G6	*HOXA9/10* (n = 6) **Genetic driver alterations reported within subtype:** 6/6 *HOXA* translocation	Given the variety of NGS approaches used and cohorts analyzed in the four genomic landscape publications the “*HOXA*” classification captures several disease subtypes. ETP-like, *MLLT10*, and *KMT2A* cases have been included in some instances resulting in variably reported outcomes
*TLX3*	17% [15], 13% [14], 8% [16], 17% [17]	Variably reported [105, 106] and likely dependent on co-occurring alterations [17]	Hypomethylating agents and venetoclax [103]. Sorafenib or quizartinib (if *FLT3* alteration present) [25, 88]	*TLX3* (n = 212) **Genetic driver alterations reported within subtype:** 181/212 *TLX3 BCL11B* enhancer; 23/212 *TLX3* fusion; 1/212 *ETV6* fusion; 1/212 *STIL::TAL1*; 1/212 *TAL1* enhancer SNV/Indel; 1/212 *LMO2* TCR; 1/212 *LMO2* intergenic loss; 2/212 unknown	*TLX3* (n = 46) **Genetic driver alterations reported within subtype:** 13/46 *TLX3* fusion; 3/46 *TLX3* copy number loss; 5/46 *TLX3* copy number gain; 1/46 *TLX3* missense alteration	G7	*TLX3* (n = 12) **Genetic driver alterations reported within subtype:** 12/12 *TLX3* translocation; 11/12 *BCL11B* translocation	
*TLX1*	10% [15], 5% [14], 18% [16], 5% [17]	Low in pediatric [15, 17] Intermediate-high in AYA/adult cohorts [104]	Hypomethylating agents and venetoclax [103]	*TLX1* (n = 71) **Genetic driver alterations reported within subtype:** 49/71 *TLX1* TCR; 13/71 *TLX1* intergenic loss; 7/71 *TLX1* intergenic inversion; 1/71 unknown	*TLX1* (n = 26) **Genetic driver alterations reported within subtype:** 6/26 *TLX1* copy number loss; 17/26 *TLX1* fusion	G8	*TLX1* (n = 23) **Genetic driver alterations reported within subtype:** 23/23 *TLX1* translocation; 5/23 *BCL11B* mutation; 1/23 *LEF1* mutation; 1/23 *NUP214::ABL1*	
*NKX2-1*	5% [15], 4% [14], 5% [17]	Low [15, 17]	-	*NKX2-1* (n = 77) **Genetic driver alterations reported within subtype:** 45/77 *NKX2-1* TCR; 13/77 *NKX2-1* chromothripsis; 7/77 *NKX2-1* intergenic loss; 4/77 *NKX2-1* fusion; 3/77 *NKX2-1* other; 3/77 unknown	*NKX2-1* (n = 14) **Genetic driver alterations reported within subtype:** 9/14 *NKX2-1* fusion; 6/14 *NKX2-1* copy number gain	G9		
*LMO1/2*	4% [15], 10% (*LMO2* only) [17]	Low in pediatric [15] and high in AYA/adult [116]. Pediatric outcomes are likely also dependent on co-occurring alterations [17]	JAK inhibitors (ruxolitinib, tofacitinib, and upadacitinib) [123], JAK2 inhibitor (fedratinib) [125]	Included in *TAL1* αβ-like and *TAL1* DP-like. *LMO1/2* alterations often co-occur with *TAL1* alterations	*LMO1/2* (n = 10) **Genetic driver alterations reported within subtype:** 4/7 *LMO2* fusion; 1/7 *LMO1* fusion; 1/7 *TAL1* noncoding alteration	G10 (*LMO1/TAL1*)		
*TAL1*	33% [15], 39% (*TAL1*/*LMO1*) [14], 8% [16], 24% [17]	Low-intermediate depending on co-occurring mutations [14, 17]	-	Comprising *TAL1* DP-like (n = 296) and *TAL1* αβ-like (n = 219) **Genetic driver alterations reported within subtypes:** ***TAL1*** **DP-like** – 78/296 *STIL::TAL1*; 66/296 *TAL1* enhancer SNV/Indel; 27/296 *TAL1* TCR; 14/296 *TAL1* enhancer gain; 11/296 *TAL1* fusion; 1/296 *ETV6* fusion; 1/296 *KMT2A* fusion; 70/296 *LMO2* TCR; 31/296 *LMO2* enhancer SNV/Indel; 10/296 *LMO2* enhancer gain; 20/296 *LMO2* intergenic loss (*RAG2*); 5/296 *LMO2* fusion; 5/296 *LMO2* intergenic loss; 2/296 *LMO2* other; 12/296 *TAL2* TCR; 5/296 *LMO1* enhancer SNV/Indel; 7/296 *LMO1* TCR; 9/296 *LYL1* fusion; 4/296 *LYL1* other; 1/296 *STAG2* other; 12/296 rare; 13/296 unknown ***TAL1 αβ-like*** - 143/219 *STIL::TAL1*; 42/219 *TAL1* enhancer SNV/Indel; 8/219 *TAL1* TCR; 7/219 *TAL1* enhancer gain; 2/219 *TAL1* fusion; 14/219 *LMO2* TCR; 43/219 *LMO2* enhancer SNV/Indel; 22/219 *LMO2* enhancer gain; 4/219 *LMO2* fusion; 8/219 *LMO2 i*ntergenic loss (*RAG2*); 2/219 *LMO2* other; 4/219 *TAL2* TCR; 22/219 *LMO1* enhancer SNV/Indel; 13/219 *LMO1* TCR; 2/219 *LMO1* other; 1/219 *LYL1* fusion; 1/219 rare; 5/219 unknown	*TAL1* (n = 87) **Genetic driver alterations reported within subtype:** 17/87 *TAL1* noncoding alteration; 58/87 *TAL1* fusion; 52/87 *TAL1* copy number loss; 1/87 *TLX3* copy number loss; 6/87 *LMO2* copy number loss; 4/87 *LMO2* fusion; 1/87 *LMO2* missense alteration; 2/87 *LMO1* fusion	G10 (*LMO1*/*TAL1*)	*TAL1* (n = 4) **Genetic driver alterations reported within subtype:** 1/4 *TAL1* translocation; 1/4 *BCL11B* mutation; 3/4 *STIL::TAL1*	Historical comparisons of this subtype are difficult given the variety of NGS approaches used. See more in-depth discussion of the *TAL1* subtypes in the text.
*TAL2*	3% [15], 1% [17]	Low [15, 17]	-	Contained within *TAL1* αβ-like and *TAL1* DP-like	*TAL2* (n = 7) **Genetic driver alterations reported within subtype:** 6/7 *TAL2* fusion; 1/7 *LMO2* fusion			
*GATA3*-mut	2% [14]	-	-			G2		As *GATA3*-mut in only captured in one dataset it is very likely a co-lesion rather than genomic driver. Pölönen et al. also reported *GATA3* alterations in several cases and defined these as a subtype driver.
*SPI1r*	1% [14], 1% [17]	High [94]	-	*SPI1* (n = 11) **Genetic driver alterations reported within subtype:** 11/11 *SPI1* fusion		G3		
*KMT2A*r	5% [17], 3% [14]	High [66], particularly poor outcomes are observed for ETP-like *KMT2A*r disease [17]	Menin inhibitors (revumenib) [70]	*KMT2A* (n = 39) **Genetic driver alterations reported within subtype:** 39/39 *KMT2A* fusion		G4		
*MLLT10*r	3% [14], 6% [16], 6% [17]	Intermediate [15] for non-ETP *MLLT10*r disease. High [42] for ETP-like *MLLT10*r disease	-	*MLLT10* (n = 31) **Genetic driver alterations reported within subtype:** 30/31 *MLLT10* fusion; 1/31 unknown		G5	*MLLT10* (n = 7) **Genetic driver alterations reported within subtype:** 7/7 *MLLT10* fusion; 1/7 *BCL11B* mutation	
*NUP98*r	*28%* [14], 2% [16], *1%* [17]	High [14, 15, 83]	-	Associated with ETP-like		Associated with G1	*NUP98* (n = 3) **Genetic driver alterations reported within subtype:** 3/3 *NUP98::RAP1GDS1*	
*NUP214*r	*28%* [14], 4% [16], *1%* [17]	High [14, 82]	-	Associated with ETP-like		Associated with G1	*SET::NUP214* (n = 7) **Genetic driver alterations reported within subtype:** 7/7 *SET::NUP214*	
*BCL11B*	8% [16], 2% [17]	Rare, outcome not available [15, 86]	Sorafenib or quizartinib (if *FLT3* alteration present) [25, 88]	*BCL11B* (n = 18) **Genetic driver alterations reported within subtype:** 10/18 *BCL11B ARID1B* Enhancer; 6/18 *BCL11B* enhancer amplication; 2/18 *BCL11B* other			*BCL11B* (n = 10) **Genetic driver alterations reported within subtype:** 5/10 *BCL11B* translocation; 5/10 *BCL11B* enhancer amplification	
*NKX2-5*r	<1% [17]	Rare, limited outcome data [17]	-	*NKX2-5* (n = 8) **Genetic driver alterations reported within subtype:** 8/8 *NKX2-5* fusion				
Tumor microenvironment-enriched	<1% [17]	Not reported [17]	-	TME-enriched (n = 42) **Genetic driver alterations reported within subtype:** 1/42 *SET::NUP214*; 1/42 *MLLT10* fusion; 3/42 *HOXA9* TCR; 7/42 *TLX3 BCL11B* enhancer; 1/42 *TLX3* fusion; 1/42 *TLX1* other; 1/42 *NKX2-1* fusion; 3/42 *TAL1* enhancer SNV/Indel; 1/42 *LMO2* fusion; 2/42 rare; 23/42 unknown				
*STAG2/LMO2*	<1% [17]	Rare, high [120]	JAK inhibitors (ruxolitinib, tofacitinib, and upadacitinib) [123], JAK2 inhibitor (fedratinib) [125], PARP-inhibitors [120]	*STAG2/LMO2* (n = 8) **Genetic driver alterations reported within subtype:** 1/8 *LMO2* TCR; 1/8 *LMO2* enhancer SNV/Indel; 1/8 *LMO2* fusion; 4/8 *LMO2* other; 6/8 *STAG2* other; 1/8 rare				
*LMO2* γδ-like	<1% [17]	High [17]	-	*LMO2* γδ-like (n = 10) **Genetic driver alterations reported within subtype:** 2/10 *LMO2* enhancer SNV/Indel; 3/10 *LMO2* fusion; 2/10 rare; 3/10 unknown				
ETP-like	18% [17]	Intermediate-high depending on co-occurring mutations [29, 32–38]	Sorafenib or quizartinib (if *FLT3* alteration present) [25, 88]; menin inhibitors for *KMT2A* ETP-like disease) [70]	ETP-like (n = 235) **Genetic driver alterations reported within subtype:** 1/235 *BCL11B* other; 16/235 *ETV6* fusion; 4/235 *ETV6* other; 14/235 *ZFP36L2* fusion; 6/235 *ZFP36L2* other; 43/235 *MED12* mutation; 23/235 *HOXA13* fusion; 11/235 *HOXA13 BCL11B* enhancer; 11/235 *HOXA13* TCR; 14/235 *NUP98* fusion; 17/235 *SET::NUP214;* 26/235 *KMT2A* fusion; 43/235 *MLLT10* fusion; 1/235 *TAL1* enhancer SNV/Indel; 1/235 *LMO2* enhancer SNV/Indel; 1/235 *LMO2* fusion; 1/235 *LMO2* intergenic loss (*CAPRIN1*); 1/235 *LMO2* other; 14/235 rare; 13/235 unknown				
**Additional frequently altered pathways**								
NOTCH1 dysregulation including alteration to the *NOTCH1*, and *FBXW7* genes	79% [15], 75% [14]	Low-intermediate depending on treatment regimen and co-occurring alterations [7, 79–81]	Potential for future NOTCH1 inhibitors but currently limited by severe toxicities [19, 20]	Associated with *MLLT10*, *HOXA9 TCR*, *TLX3*, *TLX1*, *NKX2-1*, and *TAL1* DP-like	Associated with all subtypes	Associated with all subtypes	Associated with *TLX1*, *TLX3*, *TAL1*, *HOXA9/10*, *SET::NUP214*, *MLLT10*, T-ALL rare, and G1	
JAK-STAT dysregulation including alteration to the *JAK1*, *JAK2*, *JAK3*, *STAT5A/B*, *IL7R* and *PTPN2* genes	25% [15], 21% [14]	High [14, 15]	JAK inhibitors (ruxolitinib, tofacitinib, and upadacitinib) [123], JAK2 inhibitor (fedratinib) [125]	Associated with *BCL11B*, *TLX3*, ETP-like, and *HOXA9* TCR subtypes	Associated with *LMO2*/*LYL1*, *HOXA*, *TLX3*, *TLX1*, and unknown	Associated with G1, G2, G5, G6, and G7	Associated with *BCL11B*, G1	
RAS dysregulation including alteration to the *NRAS*, *KRAS*, *PTPN11* and *NF1* genes	14% [15], 20% [14]	Intermediate [14, 15], may contribute to relapse	-	Associated with ETP-like and *SPI1*r	Associated with *LMO2*/*LYL1* and unknown	Associated with G1, G3, and G7	Associated with G1	
PI3K-AKT-mTOR dysregulation including alteration to the *PTEN*, *PTPN2*, *PIK3CD*, *PIK3CA*, *PIK3R1*, *AKT1* and *MTOR* genes	29% [15], 22% [14]	High [14, 15]	idelalisib	Associated with *TAL1* αβ-like (when wild-type *NOTCH1* and PI3K pathway alterations are present) and *NKX2-5*r	Associated with *TAL1*, *TAL2*, and unknown	Associated with G10	Associated with *TLX1*	
Cell cycle/apoptosis dysregulation including alteration to the *CDKN2A/B* and *CDKN1B* genes	-84% [15], 21% [14]	High [14, 15]	CDK4/6 inhibitors palbociclib, ribociclib, and abemaciclib [57]	Associated with *TLX1* and *TAL1* DP-like	Associated with all subtypes	Associated with G2	Associated with *TLX1*, *TLX3*, *TAL1*, *HOXA9/10*, *MYB*, T-ALL rare, and T-ALL NOS	
Ribosome (*RPL10*) – associated with *TAL1* DP-like and defines *TAL1* DP-like *RPL10* genetic subgroup	13% [15]	Low [15]	-	Associated with *NKX2-1* TCR and *TAL1* DP-like	Associated with *NKX2-1*			
Oncogene/tumor suppressor including *MYCN* and *RB1*	84% [15]	Low [15], but outcome data is limited	-	Associated with *TAL1* αβ-like, *LMO2* γδ-like, and *NKX2-5*r	Associated with all subtypes			

Note: The G1 subtype in Dai is enriched for *SET::NUP214* and *NUP98*r cases. Percentages have been italicized where alterations are associated with a subtype rather than those alterations defining the subtype outright.

*The supplementary information for this paper is limited making it difficult to interrogate genetic drivers. As such, inclusion of genetic drivers for this paper is not possible.

**Table 2:** Number of patients in each of the genomic subtypes reported in T-ALL genomic landscape publications.

Subtypes	Number reported in subtypes						
	Pediatric & AYA			Adult			Not specified
***bHLH*r**	Liu et al [15]	Müller et al [16]	Pölönen et al [17]	Liu et al [15]	Müller et al [16]	Pölönen et al [17]	Dai et al [14]
**Total patients**	264	69	1309	–	62	–	707
*TAL1*	87	3	314	–	1	–	^*#^278 (*TAL1/LMO1*)
*TAL2*	8	–	14	–	–	–	–
*LYL1*	18 (*LYL1/LMO2*)	^*^8 (*LYL1/LMO2*)	–	–	^*^19 (*LYL1/LMO2*)	–	^*#^199 (*LYL1/LMO2*)
**Chromosomal rearrangements**							
*SPI1*r	–	–	10	–	–	–	10
*KMT2A*r	–	–	65	–	–	–	18
*MLLT10*r	–	7	75	–	–	–	23
*NUP214r*	–	5	17	–	2	–	–
*NUP98r*		2	14	–	1	–	–
**Homeobox**							
*TLX1*	26	14	71	–	9	–	36
*TLX3*	46	10	223	–	2	–	^^^92
*NKX2-1*	14	–	71	–	–	–	29
*HOXA*	33	4	45 (*HOXA13*), 24 (*HOXA9*)	–	2	–	11
**Lim-domain only**							
*LMO1/LMO2*	^*^10 (*LMO1&2)*	–	131 (*LMO2*), 18 (*LMO1*)	–	–	–	–
**ETP-ALL & near ETP-ALL**	^&^44	^&^3	–	–	^&^6	–	–
**Other alterations**							
*MED12*	–	–	43	–	–	–	–
*ZFP36L2*	–	–	21	–	–	–	–
*BCL11B*	–	3	19	–	7	–	–
*MYB*	–	2	189	–	2	–	–
**Unknown**	22	6	64	–	14	–	–
**Rare**	–	5	78	–	3	–	11

*individual patients for each alteration in the subtype not listed.

- *data not reported in publication.*

*# age group unknown for 3 patients.*

^ *age group unknown for 1 patient.*

*& not included in reported total.*

*Note: ETP-ALL patients were identified in multiple subtypes in each study and do not contribute to total patient numbers. For the Liu et al study, ETP status was available for 72% of patients with the majority of ETP-ALL patients belonging to the LYL1/LMO2 subtype and the majority of near-ETP-ALL patients belonging to the TAL1 subtype. For the Dai et al study, ETP-ALL status was available for 39% of patients with the majority belonging to the LYL1/LMO2 subtype. For the Müller et al study, ETP status was available for 100% of patients with the majority belonging to the G1 subtype (LYL1/LMO2 dysregulation)*.

*Note: In Müller et al, MYB alterations were identified in low numbers despite comprising a separate subtype. Additionally, the MYB alterations identified by Pölönen et al demonstrated no subtype specificity. Potential subtypes such as MYB identified in small numbers require validation in additional cohorts before consideration as distinct subtypes*.

In addition to the variations in reported subtypes due to analysis techniques employed, different genomic subtypes occur at varying frequencies in different age groups, making the correlation of outcome data and leukemic driver alterations difficult. Here, we draw upon these four key articles and synthesize the genomic landscape of T-ALL to unravel this complexity. We describe the most common T-ALL genomic alterations identified in two or more of the genomic landscape papers as well as several additional high-risk alterations and gene fusions. Finally, we discuss potential targeted therapies, speculate on their clinical use and interrogate survival outcomes and potential targeted treatments.

## The old school: subtype classification based on immunophenotype and cytogenetics

### Immunophenotyping identifies Early T-cell Precursor ALL (ETP-ALL)

Flow cytometry is an extremely rapid and effective method of diagnosis. T-ALL is usually identified using a combination of markers, though cytoplasmic CD3 (cCD3) is almost always present. The European Group for the Immunologic Classification of Leukemia previously described 4 of the most common patterns of expression: pro-T (cCD3^+^, sCD3^−^, CD1a^−^, CD2^+^, CD5^−^, CD7^+^, CD34^−^), pre-T/immature (cCD3^+^, sCD3^−^, CD1a^−^, CD2^+^, CD5^+^, CD7^+^, CD34^−^), cortical T (cCD3^+^, sCD3^+/−^, CD1a^+,^ CD2^+^, CD5^+^, CD7^+^, CD34^−^), and mature-T (cCD3^+^, sCD3^+^, CD1a^−^ CD2^+^, CD5^+^, CD7^+^, CD34^−^) [26]. Most importantly, flow cytometry can identify ETP-ALL. ETP-ALL is defined by an immunophenotype of sCD3^-^, CD7^+^, CD1a^-^, CD8^-^, CD5^-/dim^ ( < 75%), and positive for one or more myeloid (CD11b, CD13, CD33, CD117) or stem cell (CD34, HLA-DR) antigens; expression of cytoplasmic CD3 is also often observed [3, 27, 28]. Near ETP-ALL cases fulfil the immunophenotypic requirements for ETP-ALL but have CD5 positivity [29]. Reported outcomes of ETP-ALL are varied. Early studies demonstrated poor overall and event free survival in pediatric [27, 30] and adult [28, 31] patients. However, up-front risk stratification to more intensified chemotherapy regimens has improved outcomes, particularly in pediatric patients [29, 32–38] highlighting the importance of early identification of ETP-ALL status. Interestingly, the type of events differ for ETP-ALL patients compared with non-ETP-ALL patients, with the former more likely to experience refractory disease while non-ETP-ALL patients are more likely to relapse [17]. Two studies have noted that immunophenotypically-confirmed ETP-ALL cases were enriched in the *LYL1/LMO2* subtype compared with all other subtypes (37% [15] and 44% [14]). ETP-ALL cases also demonstrated alterations in *HOXA13* and *MED12* with *ETV6* or *ZFP36L2* gene fusions (42% of ETP-ALL cases, 12% of all cases) or *HOXA9/10/11* deregulation driven by *NUP98/NUP214/KMT2A/MLLT10* gene fusions (30% of ETP-ALL cases, 7% of all cases) [17]. *BCL11B* driven T-ALL has been reported as almost exclusively in ETP-ALL cases [17]. It should be noted that alterations to several of these driver genes (*HOXA13*, *MED12*, *ETV6*, *ZFP36L2*) have also been observed in near-ETP and non-ETP cases. These observations resulted in delineation of the ETP-like subtype enriched for cases with ETP-ALL and diverse genomic driver alterations to genes involved in HSC development [17]. The ETP-like subtype is discussed in greater detail throughout this review.

Overall, these data indicate ETP-ALL can be effectively managed by contemporary treatments in all age cohorts. However, additional factors, such as remission status [38], may influence ETP-ALL patient outcome and requires further investigation.

## Cytogenetics: Karyotype and FISH-detectable rearrangements

Cytogenetics is used by diagnostic laboratories to detect chromosomal abnormalities in ALL patient cells [23]. A detailed description of the more common cytogenetic abnormalities has already been described [39]. As previously mentioned, cytogenetic karyotyping employs G-banding to detect gross chromosomal abnormalities while FISH detects deletions and chromosomal translocations e.g. *CDKN2A/B* deletions and *KMT2A* or *ABL1* rearrangements [23]. Subtype defining chromosomal rearrangements, some detectable by FISH/cytogenetic testing, are reported in three of the four genomic landscape papers and involve rearrangement of *KMT2A* [14, 17]*, MLLT10* [14, 16, 17], *SPI1* [14, 17]*, NUP214/98* [14, 16], and *ABL1* [16, 17], though, in T-ALL, only *KMT2A, ABL1*, and *MYB* rearrangements are routinely probed via FISH [23].

### Secondary co-lesions identifiable by cytogenetics

#### *ABL*-class rearrangements

Aside from *BCR::ABL1*, *ABL1* rearranged (*ABL1*r) T-ALL most typically arises from the *NUP214::ABL1* gene fusion. *NUP214* encodes a component of the nuclear pore complex responsible for transporting molecules between the nucleus and cytoplasm [40]. Cases of *NUP214::ABL1* are variably reported constituting ~1-6% of T-ALL cases depending on age group, frequently with a cortical immunophenotype and co-occur with dysregulation of *TLX1* and *TLX3* [9, 14, 16, 17, 41, 42]. Phospho-flow cytometry may be used to screen for *ABL1*r and other constitutively active kinases and to generate preliminary data on drug sensitivity. The addition of tyrosine kinase inhibitors (TKIs) such as dasatinib to intensive chemotherapy regimens in children with newly diagnosed *ABL1*r B-ALL has resulted in markedly improved outcomes compared with chemotherapy alone [43]. However, as the use of TKIs in *ABL1*r T-ALL patients has not been as extensively trialed and the benefit is not as clear, TKIs are not currently included in standard therapeutic regimens in T-ALL even when an ABL-class rearrangement is present. Asciminib, an allosteric inhibitor used to treat *BCR::ABL1*+ leukemias [44], has demonstrated in vitro [45] and in vivo [46] efficacy for *NUP214::ABL1* ALL as long as the ABL1 SH3 domain is present, supporting use of asciminib in *NUP214::ABL1* ALL [45, 46].

#### *CDKN2A/B* copy number variations

Another common alteration observed by cytogenetics involves chromosome 9, indicative of *CDKN2A/B* deletions. The *CDKN2A/B* genes encode cyclin dependent kinase inhibitors that provoke G1 and G2 cell-cycle arrest by blocking degradation of p53, suppressing oncogenesis and inducing apoptosis [47]. *CDKN2A/B* deletions frequently co-occur and are present in 39-78% of all T-ALL cases [15, 48–52]. Functional inactivation of *CDKN2A* via deletion of tumor suppressor regions are reported in >70% of *CDKN2A*^alt^ patients [53, 54]; whole gene deletion is reported in as many as 42% of *CDKN2A*^alt^ patients [55]. Unlike *CDKN2A, CDKN2B* inactivation occurs via methylation (51%) and deletion (37%) however, no significant difference in outcome has been reported for the two modes of *CDKN2B* inactivation [55]. Prognosis of patients with *CDKN2A/B* deletions in T-ALL are variable and likely depend on co-occurring driver alterations. *CDKN2A/B* alterations conferred slightly inferior prognosis in one study (5-year OS ~ 95% *CDKN2A/B*^del^ (*n* = 207) vs. 100% non-*CDKN2A/B*^del^ (*n* = 57; *p* = 0.0466 NCT00408005), though overall outcomes remained excellent [15]. Similarly, an adult T-ALL patient series reported a 2-year OS of 19% in *CDKN2A*^del^ cases (*n* = 23), vs 47% in non-*CDKN2A*^del^ patients (*n* = 78; *p* = 0.032 treated with either the CALLG2008 or MDACC Hyper-CVAD program) [56].

*CDKN2A/B* alterations promote cell cycle progression by phosphorylation of retinoblastoma protein, allowing progression from G1 to S phase [57]. Kinases CDK4/6 are involved in the transition of G1 to S phase and are attractive targets for inhibition [57]. The CDK4/6 specific inhibitors palbociclib (breast cancer phase III trial NCT01942135), ribociclib (melanoma phase II trial NCT01719380), and abemaciclib (breast cancer phase Ib trial NCT02057133) all potently inhibit CDK4/6 [57] and are potentially useful in acute leukemias with *CDKN2A/B* alterations [58].

#### *MYB*

*MYB* is a transcription factor essential for T-cell lineage commitment. In T-ALL, alterations to *MYB* constitute 19% of pediatric/adolescent young adult patients, most commonly presenting as amplification (57%), mutation (22%), and rearrangement (19%) [15]. An early report defined the *TCRB::MYB* fusion as a T-ALL subtype enriched in children aged ~2 years, though specific outcome data were not specified [15, 59]. Conversely, while more recent reports have also identified *MYB* rearrangements [14–17, 60], only one study classified *TCRB::MYB* as a subtype-defining event [16]. Patients with *TCRB::MYB* fusions frequently harbored co-occurring *CDKN2A* deletions and *NOTCH1* mutations [15, 59]. *MYB* alteration did not significantly affect 5-year OS (94% *MYB*^alt^ *n* = 49 vs. 96% *MYB*^wildtype^ *n* = 215; *p* = 0.12 NCT00408005) [15].

### Driver lesions identifiable by cytogenetics

#### *KMT2A*-rearranged (*KMT2Ar*) disease

*KMT2A*r disease is an established subtype of T-ALL and is the only defined subtype identifiable by cytogenetics [14, 17]. It is estimated that 4-8% of T-ALL patients harbor a *KMT2A* gene fusion and given *KMT2A* fusions are detectible via cytogenetics, a subtype can be readily defined for 1:12-1:25 patients [8, 42, 61]. *KMT2A* plays an essential role in regulating gene expression, notably of *MEIS1* and the *HOXA* gene cluster, which have roles in hematopoietic cell proliferation [62–64]. *KMT2A* rearrangements involve a variety of fusion partner genes though the most common observed in T-ALL include epigenetic regulators *MLLT1* and *MLLT4*, and less commonly, *MLLT3* and *MLLT10* [65–67]*. KMT2A*r T-ALL transformation reportedly occurs late in T-cell development and traditionally confers a mature immunophenotype [14]. However, Pölönen et al. characterized a subset of *KMT2A*r disease within the ETP-like subtype. In ETP-like *KMT2A*r ALL, cases demonstrate maturational stages from HSPC up to pre-T differentiation. Additionally, ETP-like *KMT2A*r ALL typically harbors *KMT2A::MLLT4* fusions whereas non-ETP-like *KMT2A*r cases exclusively contain *KMT2A::MLLT1* fusions [17]. Furthermore, ETP-like *KMT2A*r disease displayed a higher frequency of alterations to *ETV6*, *GATA3*, *IKZF1*, *RUNX1* or RAS signaling and a decreased number of alterations within the NOTCH signaling pathway [17].

ALL driven by* KMT2A* fusions is considered high-risk with dismal outcomes. In general, *KMT2A*r patients have a 6.7 fold worse overall survival compared with age-matched non-*KMT2A*r T-ALL patients [68]. Deeper interrogation of the ETP-like subtype revealed ETP-like *KMT2A*r ALL patients have adverse outcomes whereas non-ETP-like *KMT2A*r patients have higher MRD but more favorable outcomes, demonstrating heterogeneity of outcome even within the *KMT2A*r subtype [17]. Broadly, *KMT2A*r ALL constitutes 15% of adult cases [69] though current relevant outcome data is limited, especially in a T-ALL context. In all age cohorts remission is usually achieved though rapid relapse often occurs which has been postulated to result from a positive selection environment caused by chemotherapy treatment [66]. The introduction of menin inhibitors may improve *KMT2A*r patient outcomes; *KMT2A* interacts with menin after rearrangement, demonstrating menin’s utility as a pharmacological target [70]. Revumenib selectively targets menin-KMT2A interactions and phase I clinical data from relapsed/refractory patients aged 1-79 demonstrated an overall response rate of 53% (overall response encompassed complete remission inclusive of incomplete hematologic recovery, incomplete platelet recovery, and morphologic leukaemia-free state) (NCT04065399) [70].

## The new school: subtype classification based on next-generation sequencing (NGS)

NGS, incorporating WTS, WES, and WGS, is not currently part of routine diagnostic testing for many patients and is predominantly a research endeavor, though this landscape is changing rapidly and targeted NGS gene panels are often used in a diagnostic setting in some parts of the world [71, 72]. Recent studies have emphasized the importance of genomic data for risk stratification in pediatric and AYA cohorts [73–75]. Furthermore, an NGS-based classifier was recently described for T-ALL patients that is independently prognostic in both adult and pediatric cohorts, demonstrating the incorporation of NGS into diagnostic risk stratification [76]. NGS can be subdivided into WTS, WES, and WGS [77]. WTS is the most frequently utilized sequencing platform and provides clinically relevant data [78]. In short, WTS reads transcribed messenger RNA, WES analyzes the portion of the genome that is read to create proteins (exons), and WGS sequences both exons and introns where features such as gene promoters are located [77]. WGS may prove particularly important in the context of T-ALL. As Pölönen et al. report, a high frequency of non-coding alterations occur in several subtypes, highlighting the potential of WGS in the future of T-ALL diagnosis and treatment stratification [17]. Previously, studies without WGS used transcriptomic data to define the driver alteration potentially leading to misclassification (e.g. increased expression of *LMO2* can result from *BCL11B* enhancer hijacking resulting in the rare* LMO2* γδ-like subtype (Table 1)). Of the genomic landscape papers discussed here, only Pölönen et al. and Müller et al. utilize WGS (Table 1) [16, 17]. We unravel subtypes defined by next generation sequencing and comment on their relevance to the diagnosis and treatment of T-ALL. Briefly, alterations to *NOTCH1* and *FBXW7* are frequently present in T-ALL though outcomes of patients with these alterations are variably reported [7, 79–81]. Because *NOTCH1*/*FBXW7* alterations are ubiquitous in T-ALL, and are not subtype-defining, this review does not discuss them further.

### ETP-like ALL including *NUP98*r*, SET::NUP214*, and *GATA3*

The largest T-ALL cohort with associated genomic studies has identified a broader category of ALL, termed ETP-like disease, comprising ETP, near-ETP and non-ETP cases. Rather than immunophenotype alone, these subtypes are grouped by WTS expression profiling [17]. Patients with ETP-like disease harbor multiple recurrent driver alterations affecting hematopoietic stem cell (HSC) development, including activating juxtaposition of the *HOXA13* gene to enhancer regions of *BCL11B* and *TCR*; deregulation by rearrangement of *MLLT10*, *KMT2A*, *NUP214* and *NUP98*; *MED12* loss of function alterations; *ZFP36L2* rearrangements; and alterations to *ETV6* [17].

The *SET::NUP214* fusion is often associated with an ETP-like immunophenotype ( ~ 45%) [82] and unlike *NUP214::ABL1* it is not associated with *TLX1* or *TLX3* dysregulation [14, 82]. *NUP98*r T-ALL most frequently results from the *NUP98::RAP1GDS1* fusion however, other fusion partners such as *CCDC28A, LNP1*, and *PSIP1* have been reported [15]; *NUP98* fusions were also frequently identified in patients with ETP-ALL [14, 15, 83]. More recently, both *SET::NUP214* and *NUP98*r T-ALL form distinct clusters within the ETP-like genomic subtype [17]. Generally, *GATA3* expression is elevated in T-ALL and *GATA3* mutations, most frequently to the DNA binding domain (91%), were a subtype-defining feature in one genomic landscape study [14]. Diffusion map visualization of the top 5% variance genes revealed *GATA3* mutations result in an ETP-like genomic expression profile [14]. Further investigation of these cases also revealed some crossover with other distinct genomic subtypes, suggesting gene expression is determined by stage of cell-cycle arrest and oncogenic driver [17]. As the *GATA3* subtype was only identified in one study in low numbers it is likely a co-occurring lesion rather than a driver and so grouping with other genomic alterations is of greater clinical relevance.

### *BCL11B* alterations

*BCL11B* encodes a C2H2-type transcriptional repressor zinc finger protein. Alterations to *BCL11B* are related to T-ALL development [84] and recent discoveries indicate frequent noncoding translocations and enhancer hijacking events [17]. *BCL11B* alterations define a distinct genomic subtype in some T-ALL cohorts (Table 1) and up to 94% of cases with *BCL11B* activation express an ETP immunophenotype [16, 17, 60]. Patients frequently lack mutations to *NOTCH1* (typically found in >50% of patients [85]) and *PHF6*, and lack *CDKN2A* deletions, but demonstrate high expression of *KIT* and *LMO2* [16]. Additionally, the *BCL11B*-altered subtype exhibits decreased expression of *RAG1* and *RAG2* [16] and features activating *FLT3* mutations [16, 17, 60], characteristics shared with ETP-ALL. Expression levels of *BCL11B* can be linked to clinical outcome in standard risk adult patients: *BCL11B*^low^ 5-year OS was 35% (*n* = 40 vs. 53% *BCL11B*^high^ *n* = 129; *p* = 0.02 NCT00199056, NCT0098991) [86]. No difference in pediatric patient clinical outcome has been noted (5-year OS 95% *BCL11B*^high^ *n* = 43 vs. 96 *BCL11B*^norm^ *n* = 221; *p* = 0.28 NCT00408005) [15].

*BCL11B* altered ETP-ALL is associated with *FLT3* alterations [14, 17, 87] indicating first or second generation FLT3 kinase inhibitors such as sorafenib or quizartinib as potential therapeutics in this subtype when a *FLT3* alteration is present [25, 88]. Sorafenib has demonstrated in vitro efficacy in T-ALL cells at a clinically relevant dose [89]. Sorafenib treatment in post-allogeneic stem cell transplant *FLT3*-mutated acute myeloid leukaemia (AML) patients resulted in significantly improved 2-year OS (sorafenib treated 38% *n* = 30 vs. allogenic transplant alone 9% *n* = 30; *p* = 0.0001 NCT02997202) [90] suggesting a possible application for T-ALL patients with high *FLT3* expression who have received SCT. Newly diagnosed AML patients positive for *FLT3*-internal-tandem-duplication treated with quizartinib experienced a significantly improved OS compared to placebo treatment (quizartinib treated 32% *n* = 268 vs. placebo 15% *n* = 271; *p* = 0.032 NCT02668653) [91]. In general, it remains to be investigated if ETP-like patients respond well to intensified upfront chemotherapy and whether targeted therapeutics could further improve outcomes, especially in the ETP-like subtypes with specific genomic alterations (Table 1).

### *SPI1-*rearranged disease

The *SPI1* gene encodes the PU.1 protein, an important transcription factor and master regulator of hematopoietic cell development [92, 93]. *SPI1* rearrangements (*SPI1*r) result in aberrant expression of PU.1 [92, 93]. The *SPI1*r subtype expressed an immunophenotype that was similar to that reported for *LYL1* dysregulation and included low expression of T-cell related markers (CD1a, CD2, CD3e, CD4, and CD8a) and high expression of the myeloid marker CD33, potentially indicating intensive upfront chemotherapy like that required for ETP-ALL is warranted [14]. Reported fusion partners include *TCF7, STMN1* and *YWHAE* [14, 17, 94]. *SPI1* alterations have been reported as high-risk with dismal outcomes in two pediatric cohorts [17, 94]. A study of 120 patients, of which 7 harbored *SPI1* rearrangements, noted a poor 2-year OS of ~50% [94]. Though relatively rare, secondary malignant neoplasms occur more frequently in patients harboring an *SPI1* alteration (-log_10_(*p*)=14.39), however, these were myeloid/dendritic cell neoplasms and were not associated with MRD [17].

### Homeobox (*HOX*) gene dysregulation

*HOX* genes all perform similar functions and are involved in regulation of gene expression, morphogenesis, and differentiation [95]. *HOXA* dysregulation occurs through both coding and noncoding alterations and forms distinct subtypes in the reported datasets [14–17]. Dysregulation of *HOXA* genes can result from cis mechanisms (*HOXA* translocations often involving noncoding regions of the genome e.g. enhancer hijacking events) or trans mechanisms (fusion events involving other oncogenes resulting in *HOXA* cluster activation) [96]. HOXA dysregulation frequently leads to an immature immunophenotype, likely due to the critical role of *HOXA* gene expression in early development [17, 95]. Specific outcome data for each *HOXA* family member is limited aside from *HOXA11* which is considered intermediate-risk in both pediatric (*HOXA11*^alt^ 5-year OS of 89% *n* = 18 vs. 96% *HOXA11*^norm^ *n* = 246; *p* = 0.29 NCT00408005) [15] and adult cohorts (76% HOXA11^high^ *n* = 61 vs. 45% *HOXA11*^norm^ *n* = 57 vs. 57% *HOXA11*^negative^ *n* = 168; *p* = 0.012 GMALL 5/93 & 6/99) [97]. *HOXA13* alterations in the context of ETP-like disease have recently been reported as high-risk in a pediatric/AYA cohort, exhibiting significantly worse EFS and MRD (-log_10_(*p*)=1.2 and 11.9 respectively) [17].

#### Dysregulation of *TLX1* and *TLX3*, plus co-occurrence of* PTPN2*deletion

*TLX1/TLX3* are paralogs encoding important nuclear transcription factors in embryogenesis, specifically splenogenesis and the development of some sensory neurons [98, 99]. Despite a lack of involvement in normal hematopoiesis, aberrant activation of these genes is a recurrent feature of T-ALL, frequently resulting from T-cell receptor (*TCR*) gene loci rearrangements [98, 100], other noncoding translocations, intergenic inversion and hijacking by enhancers [17]. While *TLX1* and *TLX3* genes are highly similar, *TLX*3 alterations often result in a near-ETP-ALL immunophenotype with alterations to PRC2 complex genes [101], whereas *TLX1* alterations typically result in non-ETP-ALL, indicating variation in disease pathogenesis [14, 15]. Furthermore, non-coding enhancer hijacking events also differ between *TLX1* and *TLX3*. *TLX1* was found to hijack the *LINC00592* enhancer or be activated due to TCRβ or TCRδ loci rearrangement [17]. Deregulation of *TLX3* was due to hijacking of the *BCL11B* enhancer or rearrangement to the TCRβ locus,* CDK6*, or the *MYC* enhancer [17]. *PTPN2* deletions, which result in aberrant activation of the PI3K-AKT-mTOR pathway, have specifically been observed in T-ALL cases with aberrant expression of *TLX1* and have a similar incidence in pediatric and adult cohorts [15, 102]. Despite differences in ETP- and *PTPN2* deletion-status, *CDKN2A/B* are frequently deleted and *PHF6* is frequently mutated in both *TLX* subtypes [14–17]. Recent work indicates that when mutations to *PHF6* are present, novel hypomethylating agents may have utility in combination with BCL-2 inhibitors such as venetoclax [103].

Leukemia resulting from either *TLX1* or *TLX3* dysregulation predominantly affects males regardless of age group [14–17]. Despite these similarities, outcome data for each gene varies. In AYA/adult populations, patients with increased *TLX1* expression are considered intermediate-risk, but high-risk when *TLX1* expression is decreased (3-year OS 64% in *TLX1*^high^ and 36% in *TLX1*^low^ patients vs. 48% in non-*TLX1* altered patients) [104]. The *TLX1* subtype is considered low-risk in pediatric patients [17] with a reported 5-year OS of 100% (vs. 95% non-*TLX1* altered patients) [15] and disease can be effectively managed with contemporary chemotherapy. Patients with *TLX3* dysregulation are variably reported as low-risk (5-year OS 92% *TLX3*^alt^ *n* = 31 vs. 78% *TLX3*^norm^ *n* = 100; *p* = 0.099 ALL-BFM-A 1986-2000) [105] and high-risk (5-year OS 45% *TLX3*^alt^ *n* = 20 vs. 57% *TLX3*^norm^ *n* = 72; *p* = 0.049 FRALLE-93) [106] in pediatric cohorts. These disparities may be due to differences in treatment regimens and genomic profile. Indeed, recent genomic subtyping highlighted the importance of co-occurring alterations and revealed two subsets of *TLX3* dysregulation: *TLX3*-immature patients, enriched for *WT1* alterations, *NUP214::ABL1* fusions, loss of 16q22.1, *FLT3* internal tandem duplications, and JAK pathway alterations; and *TLX3* DP-like patients expressing gain of chr14q and *LEF1* and *MYB* alterations [17]. Furthermore, the *TLX3*-immature subtype was associated with a negative prognosis whereas patients with *TLX3* DP-like disease experienced favorable outcomes [17]. Potential translation of these findings into the clinic is yet to occur but it may indicate that those patients with *TLX3* DP-like disease may benefit from a less intense upfront chemotherapy regimen.

#### *NKX2-1* and *NKX2-5*-rearranged disease

Initially defined as a subtype by Liu et al. (Table 1) [15], the *NKX2-1* gene encodes a protein that binds and promotes expression of thyroid specific genes and drives intracellular serine/glycine synthesis, important for epigenetic regulation, in T-ALL [107]. *NKX2-1* rearrangements (*NKX2-1*r) typically result in a mature CD4^+^/CD8^+^ double-positive immunophenotype [14, 15, 17], indicating leukemic transformation occurs later in T-cell development and is often observed in pediatric cases [14, 15, 17]. Long term outcome data for the *NKX2-1*r subtype are somewhat limited, however, it has been noted as a low-risk subtype [17] with good early molecular response (100% *NKX2-1*r *n* = 13 vs. 95% non-*NKX2-1*r *n* = 251; *p* = 0.39 NCT00408005) [15] and a 5-year EFS of >98% in pediatric patients if day 29 MRD is <0.01%, suggesting these patients may respond well to chemotherapy intensity reduction [17].

Only recently defined as a separate subtype by Pölönen et al. (Table 1) [17], *NKX2-5*r patients feature rearrangements to *TCR*-β/δ loci and *BCL11B* enhancer hijacking [17]. *NKX2-5*r harbors a distinct genomic expression profile compared to *NKX2-1*r, with changes noted to *WT1, NRAS, NOTCH1, RB1, LEF1*, and *PTEN* [17]. Pediatric patients with *NKX2-5* alterations are typically female and younger at diagnosis with enrichment of a myeloid gene signature [17]. These patients are noted as having increased risk of MRD compared with other subtypes, though the *NKX2-5*r subtype is rare and findings should be confirmed in additional cohorts [17]. Should these observations be confirmed, patients would benefit from diligent MRD monitoring.

### *MLLT10*-rearranged (*MLLT10*r) disease

*MLLT10*-rearrangements occur in both AML and ALL and are relatively common in T-ALL, constituting 8-10% of cases [108–110]. Like some *KMT2A*r ALL, disease immunophenotype indicates leukemic transformation occurs at a later stage of maturation with the exception of ETP-like *MLLT10*r disease which demonstrates an ETP to pre-T differentiation stage [14]. ETP-like *MLLT10*r disease commonly displayed concomitant alterations to *PSIP1*, *ETV6*, *GATA3*, *IKZF1*, *RUNX1* or RAS signaling but decreased alterations to genes involved in NOTCH pathway signaling [17]. *MLLT10* partner genes typically include *PICALM, DDX3X*, and *KMT2A* [15, 108]. *MLLT10*r T-ALL confers an intermediate- to high-risk disease phenotype in pediatric/AYA cases depending on the study, with a 5-year OS of 46% (*n* = 20 vs. 72% non-*MLLT10*r *n* = 215; *p* = 0.052 GRAALL03/05) [42] in one cohort and 83% (*n* = 13 vs. 96% non-*MLLT10*r *n* = 13; *p* = 0.07 NCT00408005) [15] in another. Disease severity and outcome is highly dependent of ETP immunophenotype, with ETP-ALL cases faring worse than non-ETP ALL patients [17, 108]. Despite this variability in outcome between the different patient cohorts, data indicate this subtype can be effectively managed with contemporary therapeutic regimens [15–17, 42].

### Basic Helix Loop Helix (bHLH) gene alterations

bHLH genes encode a diverse group of transcription factors; alterations to *TAL1*, *TAL2*, and *LYL1* are considered subtype defining in T-ALL [14–17]. bHLH alterations are currently thought to confer a favorable ALL risk profile [14, 17]. Interestingly, bHLH alterations broadly result in different gene expression profiles [14–17].

#### *TAL1* and *TAL2*

Over 30 years ago Brown et al. described a genomic alteration observed in ~25% of T-ALL cases, involving the chimeric fusion *STIL::TAL1* [111]. This observation has been recapitulated in recent patient cohorts with designation into subtypes involving *TAL1* or *TAL2* (Table 1) [14–17]. TAL1 is a bHLH transcription factor involved in mediating hematopoietic stem cell fate by regulating gene expression in megakaryocytic/erythroid progenitors [112]. TAL1 also interacts with other known T-ALL associated genes such as *LMO2*, which, under normal conditions, plays an essential role in lineage fate determination [112]. PI3K-signaling alterations are frequently enriched in *TAL1*-altered T-ALL and may be clinically targetable [17]. The fusion of *TAL1* with *STIL* is frequently due to an interstitial deletion between the two genes placing *TAL1* under the regulatory control of the *STIL* promoter resulting in aberrant super expression of TAL1, leading to malignancy [113, 114]. Observed in ~30% of childhood and adult T-ALL cases, *STIL::TAL1* requires cooperation from other genomic events involving *LMO1* or *LMO2* or activation of the NOTCH pathway [16, 53, 85]. Additionally, *TAL1* overexpression can be due to formation of a super-enhancer caused by introduction of mutations in a noncoding region upstream of the *TAL1* transcriptional start site. These mutations create de novo binding motifs for the MYB transcription factor resulting in super expression of *TAL1* [115]. Recently, WGS identified additional noncoding alterations involving *TAL1* within a pediatric cohort [17] including *TAL1* enhancer gain, enhancer alteration via SNV/Indel mutations, enhancer hijacking events, intergenic inversions and noncoding rearrangements.

*STIL::TAL1* is rare in adult T-ALL patients [14], but those with *STIL::TAL1* experience similar 4-year OS rates as those without *STIL::TAL1* (both 45%) [116] suggesting outcomes are similar with current treatment regimens. Combined with the outcome data mentioned above, survival of *TAL1* dysregulated patients varies depending on patient age, the type of *TAL1* alteration and co-occurring lesions [14, 17].

#### *LYL1*

LYL1 has purported roles in blood vessel maturation and hematopoiesis where it interacts with LMO2 [117]. *LYL1* driven T-ALL results from gene activation and was identified as a subtype defining alteration in two of the genomic landscape studies [14, 15]. In both instances *LYL1* perturbations co-occurred with *LMO2* dysregulation, likely due to established interactions between the two transcription factors [14, 15, 117]. Pediatric patients with alterations to *LYL1* are reported to have a worse EFS and MRD [17].

Overall, alterations of bHLH genes *TAL1*, *TAL2* and *LYL1* demonstrate mutual exclusivity [17]. Leukemia arising from *TAL1* dysregulation has been described in predominantly male pediatric patients [14–16]. Recent data demonstrates the importance of co-occurring genomic alterations in *TAL1* driven T-ALL [17]. Termed* TAL1*αβ-like and* TAL1* double positive (DP)-like, these subtypes differ in immunophenotype and gene alterations.* TAL1* αβ-like disease exhibits a higher frequency of *STIL::TAL1* fusions, *LMO1* enhancer SNVs, and is enriched for *MYCN* mutations [17]. Conversely,* TAL1* DP-like T-ALL has a mature immunophenotype (CD4^+^/CD8^+^), increased expression of *RAG1/2*, and increased frequency of *LMO2* TCR rearrangements [17]. Clinical utility of these subtypes is yet to be fully realized, however, recent data suggest *TAL1* αβ-like and *TAL1* DP-like patients demonstrate a significantly worse OS (-log_10_(*p*)=1.5) and disease free survival (-log_10_(*p*)=2.4) respectively compared to other subtypes [17]. If patients can be more accurately grouped based on gene expression profiles rather than individual driver alterations, then appropriate treatment regimens can be selected, potentially significantly improving patient outcomes.

### *LMO1* and *LMO2*

*LMO1* and *LMO2* encode members of a family of nuclear transcription co-regulators involved in mediating protein-protein interactions rather than binding directly to DNA [118]. *LMO2* is an essential regulator of hematopoiesis and angiogenesis, and deletion in murine models results in lethality during early development [119]. *LMO1* is a paralog of *LMO2* but has a distinct function in neurogenesis [118] and *LMO1* and *LMO2* alterations are mutually exclusive [14, 17]. Dysregulation of *LMO2* gene expression is observed in two different subtypes: combined with dysregulation of either *LMO1* [15] or *LYL1* [14, 15]. Similarly, *LMO1* dysregulation forms a distinct subset when combined with *TAL1* dysregulation [14, 15]. Activating mutations to the JAK/STAT signaling pathway co-occur in the *LYL1/LMO2* (*JAK1, JAK3*, and *STAT5A*) and *TAL1/LMO1* (*JAK1, JAK3, STAT5A, STAT5B*) subtypes [14]. Historically, AYA/adult patients with *LMO2* alterations were considered high-risk (4-year OS 29% *LMO2*^alt^ *n* = 15 vs. 44% *LMO2*^norm^ *n* = 54, LALA-94) [116] but pediatric patients low-risk (5-year OS 90% *LMO2*^alt^ *n* = 20 vs. 96% *LMO2*^norm^ *n* = 244; NCT00408005 *p* = 0.078) [15]. New insight from a pediatric cohort has identified two additional subtypes of *LMO2* termed *LMO2* γδ-like and STAG2/LMO2. Patients clustering in the *LMO2* γδ-like subtype displayed *LMO2* activation from *BCL11B* enhancer hijacking, TCRδ rearrangements, and *LMO2* enhancer SNV/Indels [17]. *LMO2* γδ-like is associated with poor outcome and has a 5-year EFS < 60% [17]. The *STAG2/LMO2* subtype typically involves a *LMO2::STAG2* rearrangement resulting in *LMO2* activation and simultaneous *STAG2* inactivation [17, 120, 121]. These patients are predominantly female, very young at diagnosis, and constitute a rare subtype of T-ALL ( < 1% of cases analyzed) [17]. Patients with *STAG2/LMO2* ALL who are <3 years old and have ≥1% MRD following induction have dismal outcomes [120].

Initial analyses by Liu et al. suggested association of *LMO1* dysregulated pediatric patients with a poor outcome [15] however, additional analyses by Pölönen et al., which include patients from the original cohort, observed no association [17]. The severity and risk of relapse from *LMO* driven disease is highly dependent on patient age, the specific driver alteration or the co-occurring lesion/s contributing to disease pathogenesis [15, 17, 116].

As previously mentioned, activating mutations to the JAK/STAT signaling pathway are frequently observed in the *TAL1/LMO1* and *LYL1/LMO2* subtypes rendering tyrosine kinase inhibitors as attractive targeted therapies [122]. In vitro studies of the JAK inhibitors ruxolitinib, tofacitinib, and upadacitinib all significantly reduced growth of Ba/F3 cells harboring common JAK/STAT activating mutations such as *JAK1* p.A634D, *IL7R* ins*SRCL*, *JAK3* p.M511I, *JAK3* p.L857Q (except upadacitinib) [123]. Ruxolitinib has also demonstrated ex vivo efficacy and synergistic action when used in combination with venetoclax in patient samples harboring activating mutations to *IL7R* pathway genes [124]. The JAK2 inhibitor fedratinib is active against wild-type and mutated JAK2 and also FLT3, suggesting potential utility in leukemias harboring activating mutations in both genes [125].

## Conclusion

T-ALL is a genomically complex disease which is frequently fatal upon relapse. Here we have summarized four T-ALL genomic landscape studies and found consensus in subtype defining alterations where possible. We have also described patient outcomes and potential targeted treatments for laboratory and clinical investigation. T-ALL occurs less frequently than B-ALL and, until recently, has remained an under-studied cohort, particularly in terms of genomic interrogation of large patient datasets. The synopsis presented here highlights the genomic complexity of T-ALL and the importance of co-occurring lesions on disease progression and outcome. Of significance is the recent insight gained from WGS analyses and the advantage over traditional diagnostic techniques, particularly in the T-ALL setting. These genomic methodologies have identified the frequency at which noncoding alterations occur in T-ALL, revealing a genomic landscape predominantly comprised of noncoding lesions. Given we now know that noncoding alterations have a role as T-ALL driver lesions as well as secondary co-lesions impacting outcome, in the future, detailed genomic analyses may be required to inform the clinical approach for disease treatment and potential eradication.
